# Supplementary material for: DMRforPairs: identifying Differentially Methylated Regions between unique samples using array based methylation profiles
Source: BMC Bioinformatics. 2014 May 15;15:141. doi: 10.1186/1471-2105-15-141 (PMC4046028; doi:10.1186/1471-2105-15-141)
Supplement: Additional file 2 — DMRforPairs output for the comparison of A431-MCF7 and NA17018-NA17105. Please start from the HTML files in each folder. Available via the BMC Bioinformatics website. [file 1471-2105-15-141-S2.zip › 1394847754114233_MOESM2_ESM/A431_MCF7/figures/33590.pdf]

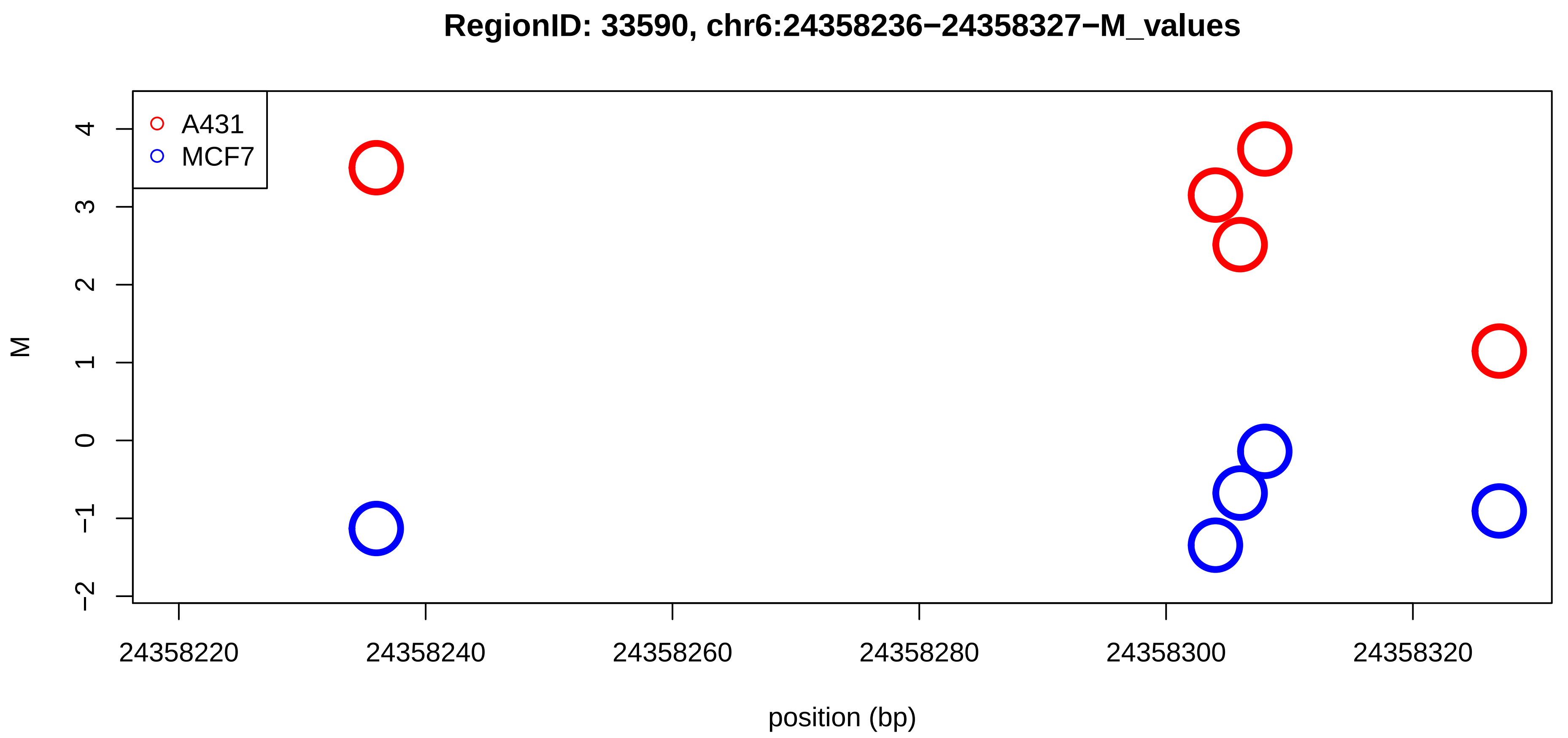
RegionID: 33590, chr6:24358236−24358327−M_values
A431
MCF7
M
4
3
2
1
0
−1
−2
24358220
24358240
24358260
24358280
24358300
24358320
position (bp)

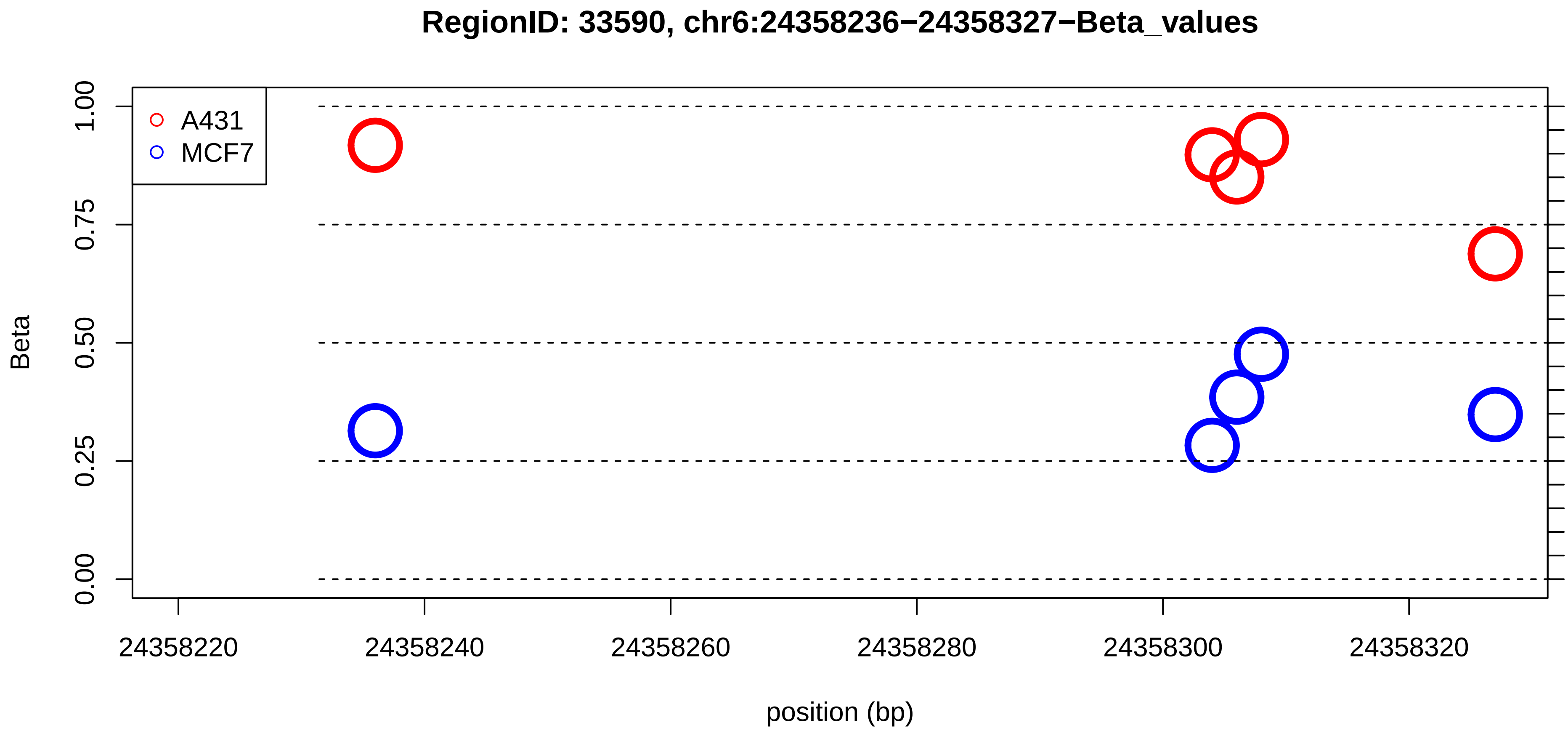
RegionID: 33590, chr6:24358236−24358327−Beta_values
A431
MCF7
Beta
1.00
0.75
0.50
0.25
0.00
24358220
24358240
24358260
24358280
24358300
24358320
position (bp)

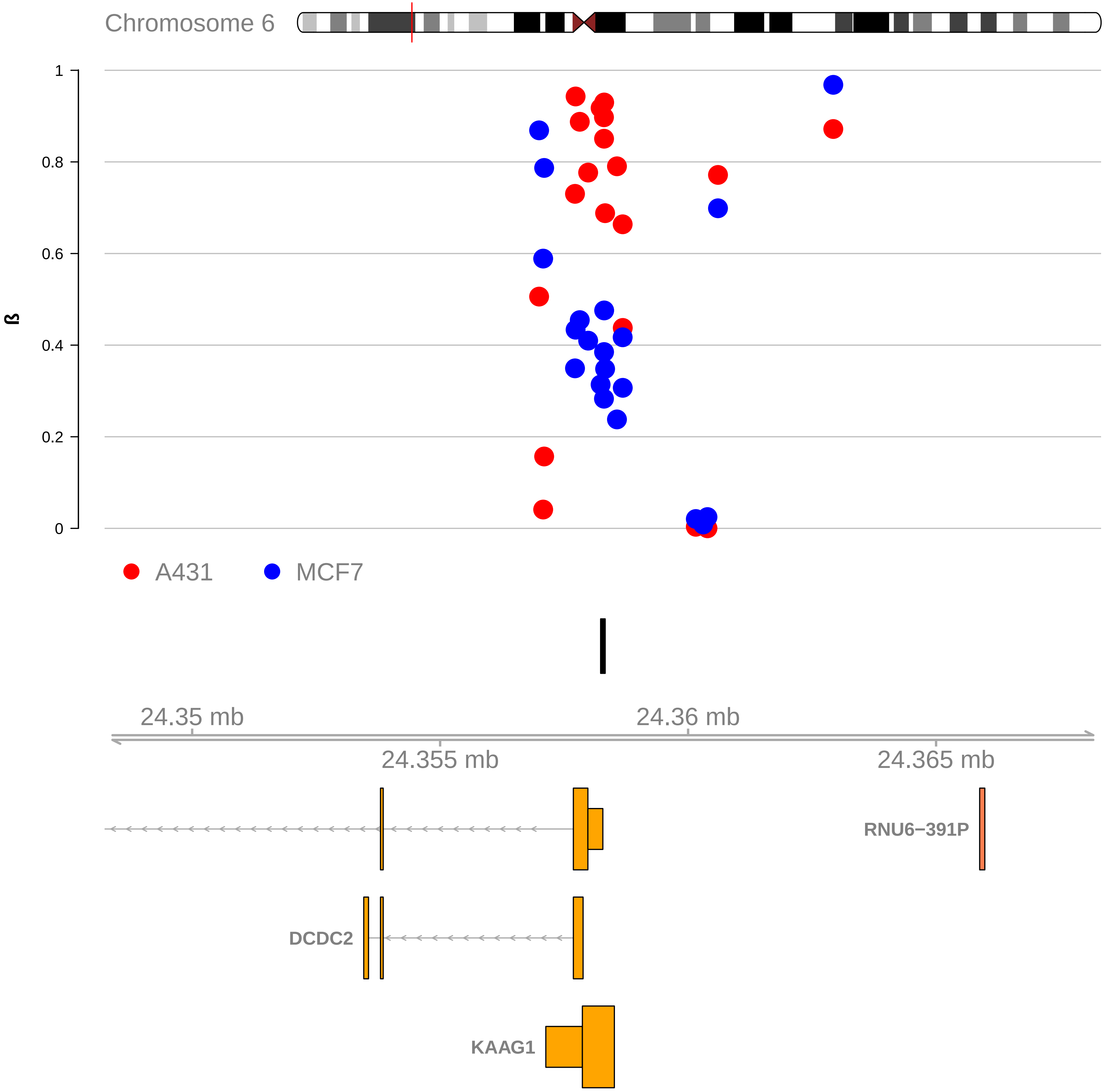

Chromosome 6
1
0.8
0.6
0.4
0.2
0
ß
A431
MCF7
24.35 mb
24.36 mb
24.355 mb
24.365 mb
RNU6−391P
DCDC2
KAAG1
